# Effects of Touch Location and Intensity on Interneurons of the Leech Local Bend Network

**DOI:** 10.1038/s41598-018-21272-6

**Published:** 2018-02-14

**Authors:** Friederice Pirschel, Gerrit Hilgen, Jutta Kretzberg

**Affiliations:** 1 Computational Neuroscience, Department for Neuroscience, University of Oldenburg, Oldenburg, Germany; 2 Department of Organismal Biology and Anatomy, University of Chicago, Chicago, IL USA; 3 Institute of Neuroscience, Newcastle University, Newcastle upon Tyne, United Kingdom; 4 Cluster of Excellence “Hearing4all”, University of Oldenburg, Oldenburg, Germany

## Abstract

Touch triggers highly precise behavioural responses in the leech. The underlying network of this so-called local bend reflex consists of three layers of individually characterised neurons. While the population of mechanosensory cells provide multiplexed information about the stimulus, not much is known about how interneurons process this information. Here, we analyse the responses of two local bend interneurons (cell 157 and 159) to a mechanical stimulation of the skin and show their response characteristics to naturalistic stimuli. Intracellular dye-fills combined with structural imaging revealed that these interneurons are synaptically coupled to all three types of mechanosensory cells (T, P, and N cells). Since tactile stimulation of the skin evokes spikes in one to two cells of each of the latter types, interneurons combine inputs from up to six mechanosensory cells. We find that properties of touch location and intensity can be estimated reliably and accurately based on the graded interneuron responses. Connections to several mechanosensory cell types and specific response characteristics of the interneuron types indicate specialised filter and integration properties within this small neuronal network, thus providing evidence for more complex signal processing than previously thought.

## Introduction

The medicinal leech responds to tactile stimulation in a highly precise manner; it bends away from the site of mechanical stimulation with surprising accuracy: The animal can behaviourally discriminate between touch locations that are only 9° (~500 µm) apart^1^. This so-called local bend response^1–10^, is sensitive to touch location, to touch intensity and duration^1,10^.

**Figure 1:**
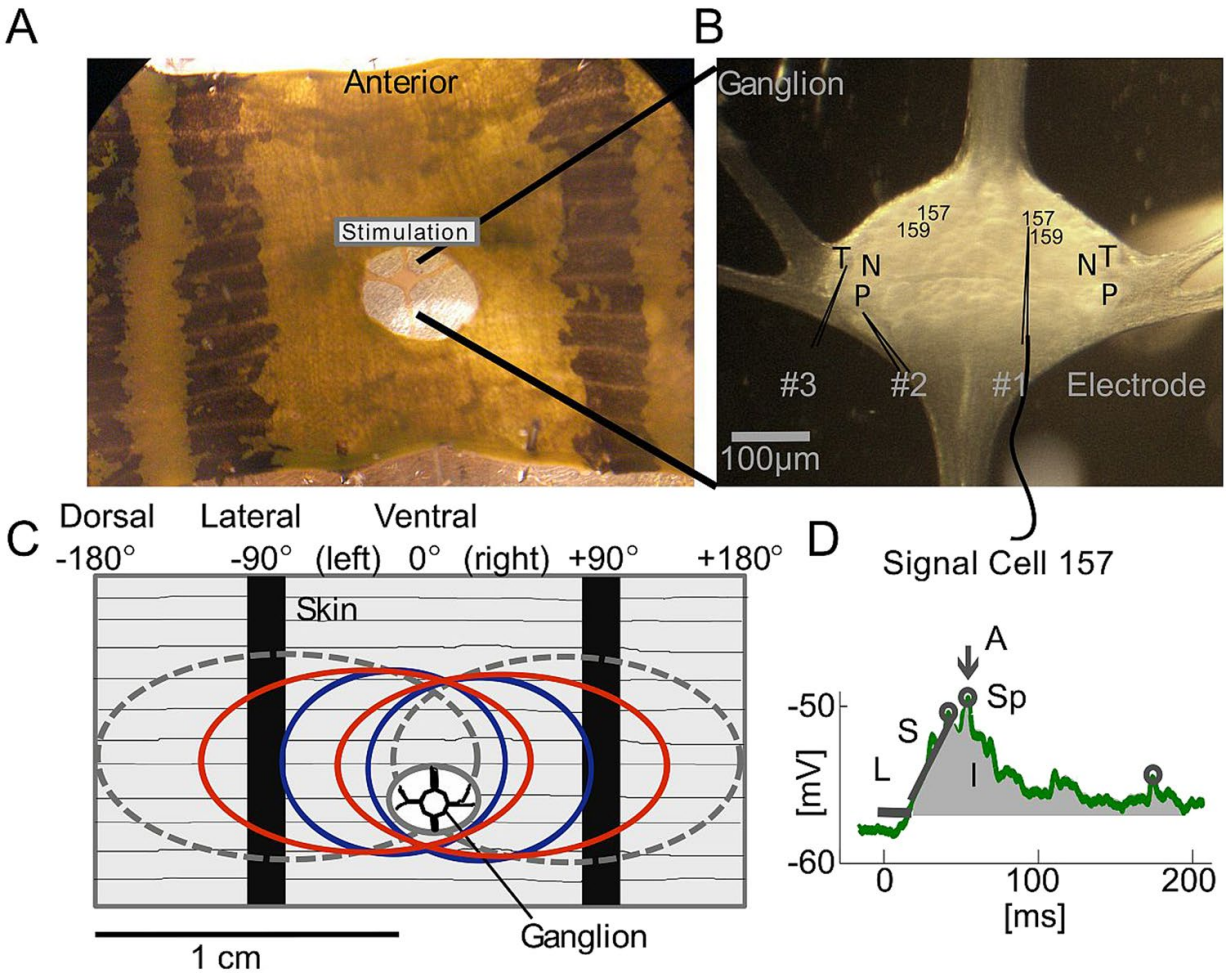
Photographs of the body-wall preparation and sketch of the receptive fields of mechanosensory cells. (**A**) Photograph shows the body-wall preparation (see *Methods*). Access to the ganglion is provided by a hole in the skin. In grey: Segmental annulus used for tactile stimulation. (**B)** Magnified ganglion with electrodes and positions of ventral T, ventral P, and lateral N cell bodies and cell 157, 159. Responses of up to 3 neurons were recorded intracellularly during mechanical skin stimulation (see *Methods*). (**C**) Ventral midline (centre line anterior-posterior between the two dark stripes on the skin) is defined as 0°. Touch locations to the right (experimenter’s perspective) were denoted as a positive number of degrees and to the left as negative number. The left end of the preparation marks −180°, the right side +180°, black stripes are at −90° and +90°. The sketch of the body wall preparation shows the approximate locations and extents of the receptive fields of all mechanosensory cells sensitive to touch at the ventral midline: two T cells (blue), two P cells (red), two N cells (dashed grey). (**D)** Sketch showing the analysed response features: Amplitude (arrow), slope (inclined line), latency (horizontal line), integral (grey area), and spikelets (circles) (see *Methods*).

The medicinal leech possesses a relatively simple and easily accessible neuronal system^11,12^ with individually identifiable, monopolar neurons^13^, and accurate behavioural patterns. Three types of mechanosensory cells with distinct receptive fields^14–19^ (see Fig. 1) are situated in each segmental ganglion of the leech: six T (touch) cells, four P (pressure) cells and four N (nociceptive) cells^14^. Additionally, each ganglion contains interneurons (INs) and motor neurons (MNs) and as a result, one isolated ganglion, with its 400 neurons in total, is sufficient for eliciting this behaviour^10,11^. Earlier studies focused on P cells as a main trigger for the local bend response, since T cells showed only minor contributions to muscle movements during the behaviour^3,9,18,20^. However, Thomson and Kristan^1^ found that electrical stimulation of two ventral P cells with overlapping receptive fields resulted in a less precise muscle movement than induced by mechanical skin stimulation. Indeed, we showed in preceding studies^21,22^ that T cells encode touch locations very precisely. These studies suggest that T cells might play a substantial role for the local bend response.

At the next network level, at least nine types of INs are known to be involved in the local bend response^5^. These neurons have synaptic connections on MNs, which elicit the muscle contraction or elongation during the local bend^4,5^. Most of the local bend INs receive input from all four P cells in one ganglion indicating that these INs are not specialised for eliciting only one local bend direction but are rather activated by a wider range of touch locations mediated by the corresponding mechanosensory cells^5^. At least some of the local bend INs also receive input from T cells^22^, but the relative contributions of the different types of mechanosensory cell inputs are not known yet.

Here, we focused on two local bend INs^5^ (cell 157 and 159) which respond with graded membrane potential changes and spikes of very small amplitude (spikelets) to synaptic inputs from mechanosensory cells. We investigate their morphological connections to mechanosensory cells and their response characteristics to touch location and intensity. We further investigated if it is possible to estimate stimulus properties based on graded response features (such as integral, amplitude, latency, and slope) or the spikelet count. Therefore, we use two complementary maximum-likelihood approaches for stimulus estimation: a pairwise discrimination of stimulus differences and a classification of all possible stimulus conditions^21^.

## Results

### Response characteristics of cell 157 and cell 159

**Figure 2:**
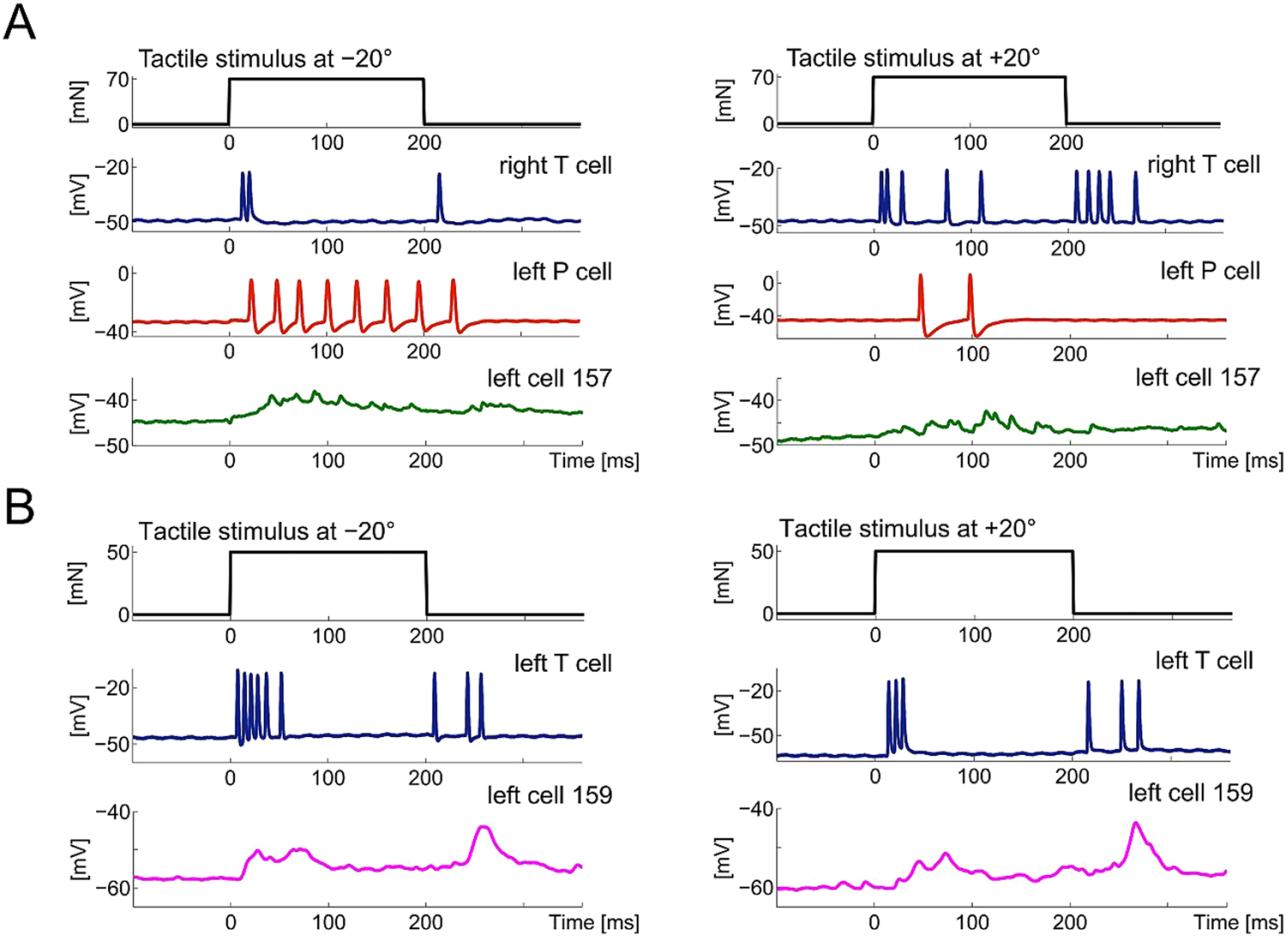
Responses of mechanosensory cells and INs to different touch locations. (**A**) Representative responses from one experiment of simultaneously recorded right T cell (blue), left P cell (red) and left cell 157 (green) to a touch stimulus (black) of 70 mN for 200 ms at locations −20° (left) and +20° (right). (**B**) Representative responses from one experiment of simultaneously recorded left T cell (blue) and left cell 159 (magenta) to a touch stimulus (black) of 50 mN for 200 ms at locations −20° (left) and +20° (right).

Little is known about the response patterns of local bend INs to naturalistic stimulation or about their connections to mechanosensory cells other than P cells. Cell 157 and cell 159 responded with distinct excitatory PSPs (EPSPs) to tactile skin stimulation (Fig. 2). The EPSP in cell 157 increased in the first 50 ms after response latency and was followed by a slow decay (Fig. 2A, green). Remarkably, cell 159 showed a biphasic response with two clear EPSPs, one at the beginning and one at the end of the stimulus (Fig. 2B, magenta), the latter being more pronounced. This pattern clearly reflects the responses of T cells, which generate bursts of spikes after stimulus onset and offset^21–23^ (Fig. 2).

The examined INs showed fast spikelets^24^ of small amplitudes, approximately 2–5 mV (Figs 1 and 2), indicating that the somatic membrane does not contain a high density of voltage-dependent ion channels^5^. The frequency of these spikelets depended on the membrane potential (not shown), as also described by Lockery and Kristan^5^: Penetration of the cell membrane depolarizes the IN soma (around −20 to −30 mV somatic membrane potential) which results in frequent spontaneous spikelets. After stabilization of the membrane potential to values more hyperpolarized than −40 mV, both IN types barely show any spontaneous spikelets (Figs 1 and 2).

### Influence of touch location and intensity

**Figure 3:**
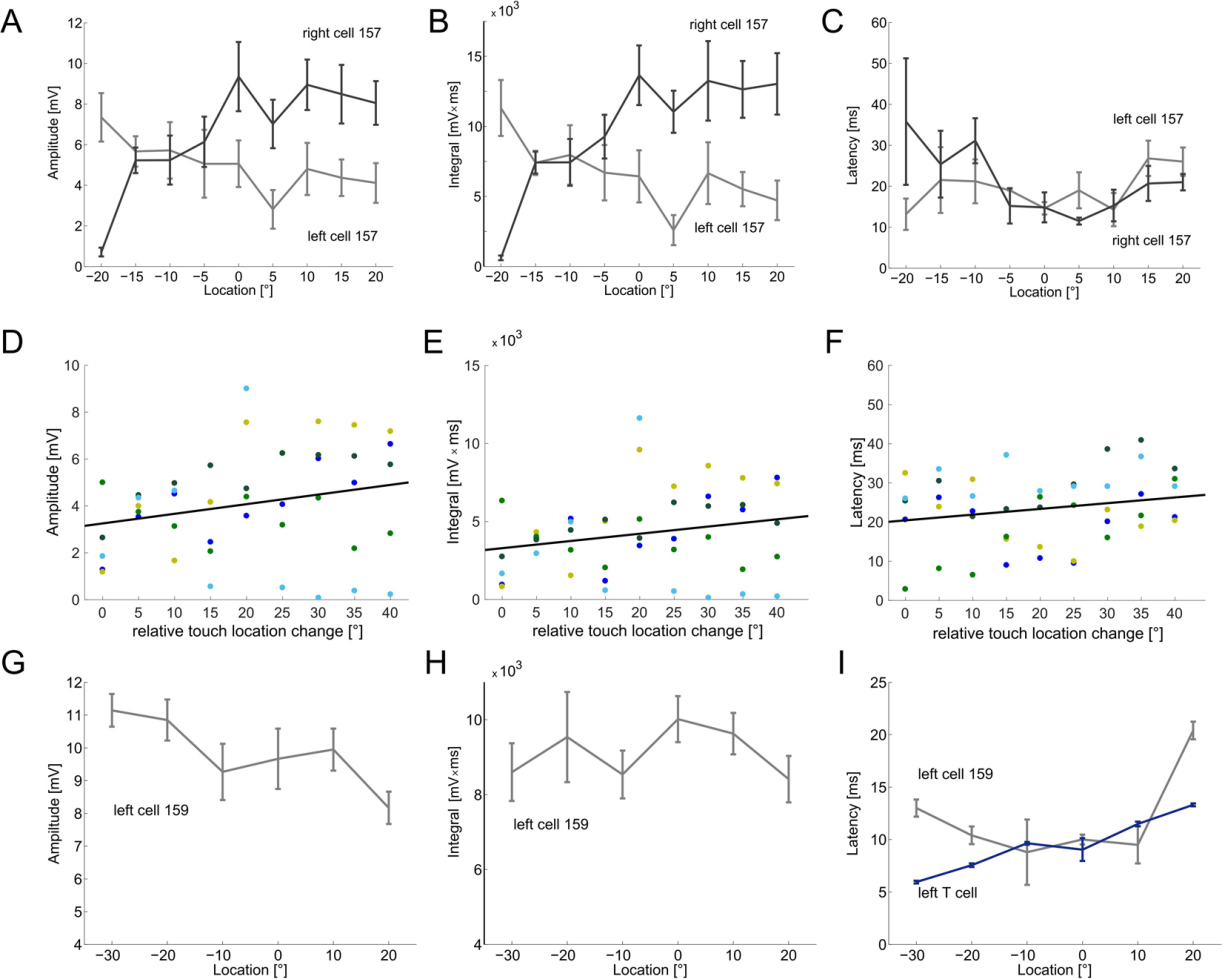
Responses of cell 157 and 159 to different touch locations. (**A**) Amplitude of EPSPs (relative to resting potential) in mV (mean and STD of 10 representations) of a right (light grey) and a left (dark grey) cell 157 stimulated with 70 mN at locations between −20° and +20°. (**B**) Integral (mean and STD) for the same data as in A. (**C**) Latency in ms (mean and STD) for the same data as in A. (**D**) Amplitude of EPSPs in mV of five cells 157 (N_cells_ = 5; 2 cells on the right side of the ganglion, marked with blue shaded dots, and 3 cells on the left side shown in green shaded dots; N_animals_ = 4) in respect to touch locations change towards receptive field centre. Here, ‘0°’ represents +20° for left cells, −20° for right cells and ‘40°’ is −20° for left cells, +20° for right cells. Black line represents the linear fit over cells (y = 0.62x + 3.42; R^2^ = 0.94). Dots represent mean values over trials (N_trials_ = 10) per cell (N_cells_ = 5). (**E**) Integral (mean over trials) for the same data as in D. Black line represents the linear fit over cells (y = 917.6x + 2553.5; R^2^ = 0.94). (**F)** Latency in ms (mean over trials) for the same data as in D. Black line represents the linear fit over cells (y = −2.1x + 36.12; R^2^ = 0.97). (**G)** Amplitude in mV (mean and STD of 10 representations) of a representative left cell 159 recording stimulated with 70 mN at locations between −30° and + 20°. (**H**) Integral (mean and STD) of the same data as in G. (**I**) Latency in ms (mean and STD) of the same data as in G (light grey) and of the simultaneously recorded left T cell (blue).

**Figure 4:**
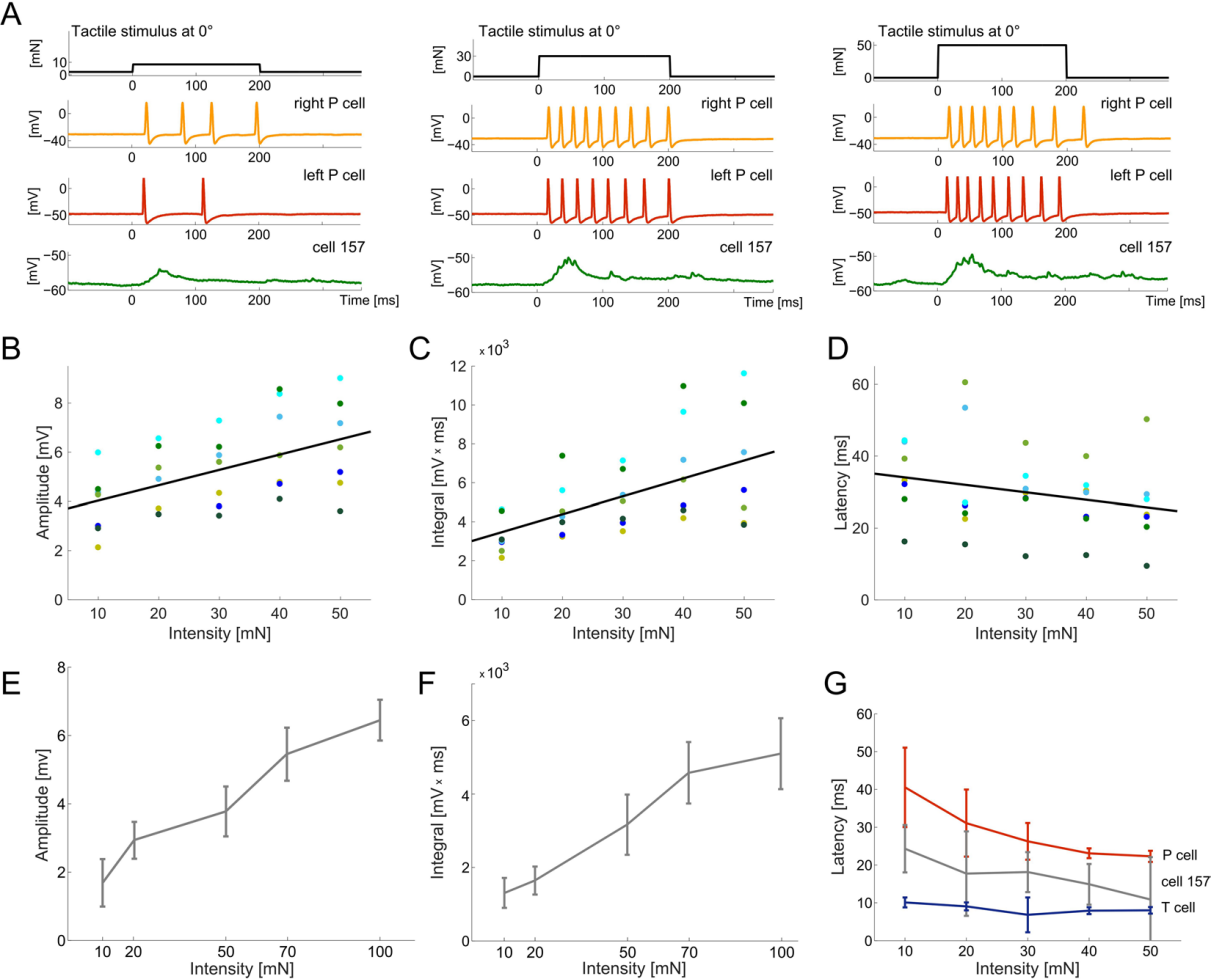
Responses of cell 157 and mechanosensory cells to different stimulus intensities. (**A**) Representative responses [mV] of simultaneously recorded right P cell (orange), left P cell (red) and left cell 157 (green) to a touch stimulus (black) of 10 mN (left), 30 mN and 50 mN (right) at 0° lasting for 200 ms. (**B)** EPSP amplitude (in mV) of seven cells 157 (N_cells_ = 7; consisting of 3 cells on the right side of the ganglion, marked with blue shaded dots, and 4 on the left side shown in green shaded dots; N_animals_ = 7) for intensities of 10 to 50 mN at location 0°. Black line represents the linear fit over cells (y = 0.21x + 3; R^2^ = 0.32). Dots represent mean values over trials (N_trials_ = 10) per cell. (**C)** Integral of the same data as in B (dots: mean values over trials, black line: linear fit over cells, y = 231.6x + 3054.4; R^2^ = 0.26). (**D)** Latency in ms of the same data as in B (dots: mean latency over trials, black line: linear fit over cells, y = 0.73x + 20; R^2^ = 0.38). (**E)** EPSP amplitude in mV (mean and STD of 10 representations) of one representative cell 157 recording for intensities of 10 to 100 mN at location 0°. (**F)** Integral (mean and STD) of the same data as in E. (**G)** Latency in ms (mean and STD) for a representative simultaneous recording of cell 157 (grey: same as in B and C), T cell (blue) and P cell (red) for intensities of 10 to 50 mN at location 0°.

We next examined how touch properties were reflected in the IN responses. All recorded cells identified as cell 157 showed an influence of the touch location on the graded response features amplitude, integral, latency (Fig. 3A–F) and slope (not shown). Each of these features depended significantly (Friedman test, p < 0.001; N_cells_ = 5) on touch location for all examined intensities. Like mechanosensory cells^21^, cell 157 seemed to have a spatially structured receptive field (Figs 2A and 3A–C) showing more pronounced EPSPs when the touch location is closer to the receptive field centre with a body-wall location ipsilateral to the IN-cell body in the ganglion (Figs 2 and 3). This result was found for cell 157 on both sides of the ganglion (Fig. 3D–F). One exemplary recording of a cell 159 on the left side of the ganglion showed a similar tendency and responded with smaller EPSPs at +20° (right of the ventral midline) and higher amplitudes at −30° (Fig. 3G,H). These results confirm previous conjectures about the IN receptive fields^7,11,19^. In addition to touch location, cell 157 responses also reflected touch intensities: higher intensities elicited significantly stronger responses (for all graded response features, Friedman test, p < 0.001; N_cells_ = 5). Amplitude and integral of cell 157 increased in a linear manner between 10 and 70 mN, while latency decreased (Fig. 4). At the ventral midline, responses of cell 157 on both sides of the ganglion depended similarly strong on touch intensity (Fig. 4B–D).

In response to tactile stimulation, the IN responses in both cell types started shortly after the first T cell spikes and before the first P cell spike occurred^21^ (Figs 3F and 4G). For different touch intensities applied at the ventral midline, the response latencies of the two mechanosensory cells (Fig. 4G; ‘P’, red; ‘T’, blue) and the cell 157 latency (Fig. 4G; grey) reveal that the IN response reliably starts earlier than the first P cell spike. We found in a previous study that, at the ventral midline, the latencies for the two ventral mechanosensory cells of one cell type (P or T cells) are equally long but with T cells having a significantly shorter response latency than P cells^21,22^. Taking this into account, it suggests that the fast and precise T cells add valuable information about the touch stimulus to the IN response.

### Morphological and physiological connections between mechanosensory cells and interneurons

**Figure 5:**
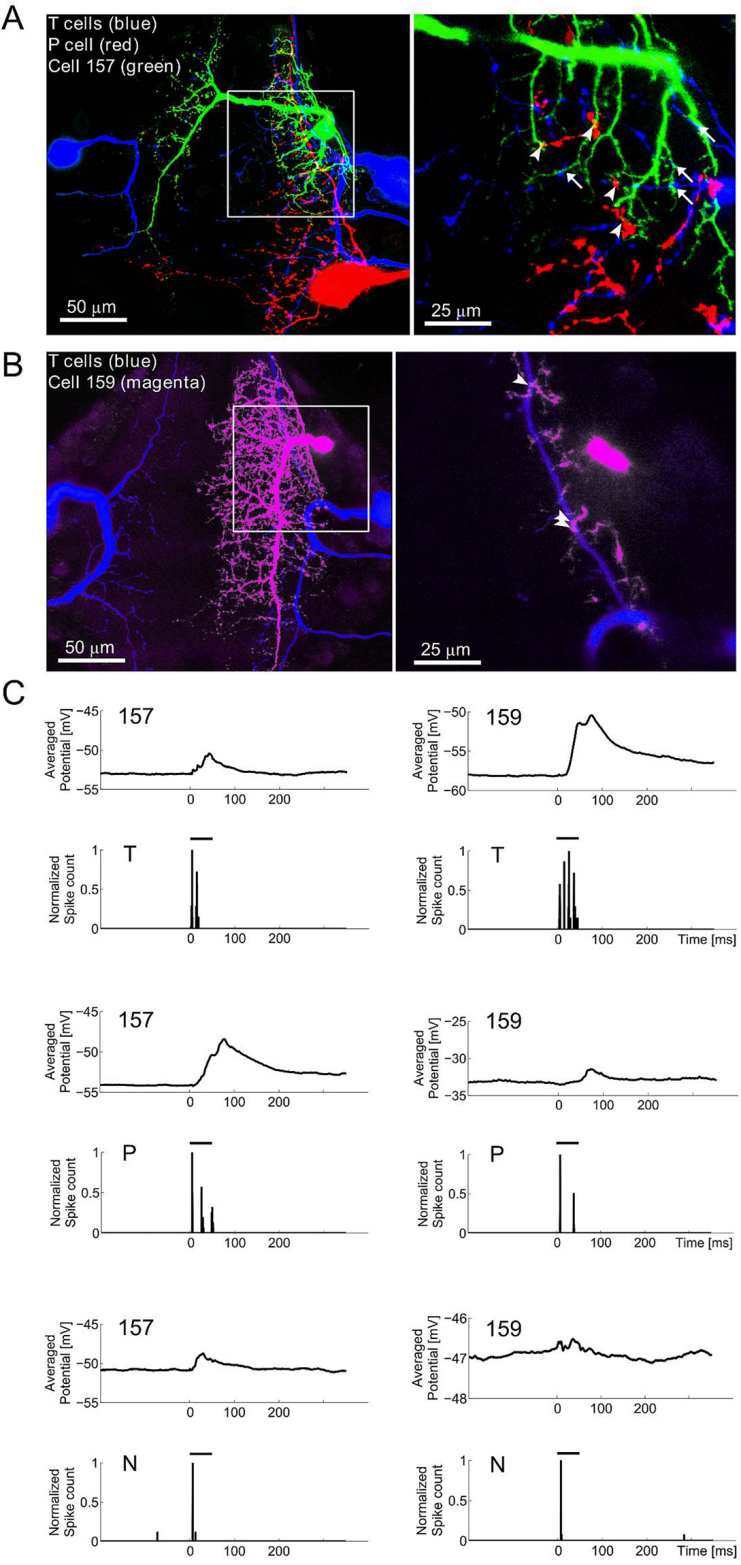
Connections between cell 157 and 159 and ipsilateral mechanosensory cells. (**A**) Cell staining of T cells (blue, filled with Alexa-dyes; see *Methods*), P cell (red, filled with Alexa-dyes) and cell 157 (green, filled with Neurobiotin). Right: magnifications of rectangle in left image with putative P cell input sites (arrowheads), and putative T cell input sites as arrows. (**B**) Cell staining of T cells (blue, filled with Alexa-dyes) and cell 159 (magenta, filled with Alexa-dyes). Right: magnifications of rectangle in left image with putative T cell input sites (arrowheads). (**C**) Paired recordings of mechanosensory cells with ipsilateral cell 157 (left side) or ipsilateral cell 159 (right). The mechanosensory cells (ventral T and P, and lateral N) were stimulated by intracellular current injection (horizontal black bar, see *Methods*). Results show means of 20–50 repeated current injection into the mechanosensory cell for the given ipsilateral cell pair. First row: T cell stimulation; Second row: P cell stimulation; Third row: N cell stimulation. Note the different y-axis scaling of IN 159 responses to T and P versus N cell stimulation.

Connections between the different mechanosensory cells (ventral P and T cells and lateral N cells) and cell 157 as well as cell 159 were analysed morphologically by intracellular dye-fills (Fig. 5A,B) and physiologically by intracellular, paired recordings using electrical stimulations of the mechanosensory cells (Fig. 5C). We found significant changes of the membrane potential of cell 157 due to P cell spikes, T cells spikes or N cell spikes in all ipsilateral recordings (Kolmogorov-Smirnov, p < 0.001; Fig. 5C, left column) as well as contralateral recordings (Kolmogorov-Smirnov, p < 0.001). Connections of T and P cells to cell 157 were also suggested by cell-specific structural imaging (Fig. 5A). Magnifications of a subset of confocal microscope layers showed putative input sites of P (Fig. 5A; red, arrowheads) as well as of T cells (Fig. 5A; blue, arrows) to cell 157 (Fig. 5A; green).

Cell 159 is located near cell 157 (Figs 1 and 5B) and showed a distinct response pattern to tactile stimulation (Fig. 2). The EPSPs in response to tactile stimulation follow the response of the T cells, which typically generate a burst at stimulus onset and a burst after stimulus offset (Fig. 2). A preliminary data set of intracellular recordings and dye-filling of cell 159 and mechanosensory cells shows responses in cell 159 consequent on T cell stimulation (Fig. 5C) as well as contact points of T cells (Fig. 5B; blue) and cell 159 (Fig. 5B; magenta). This might suggest a synaptic coupling of these two cell types (Fig. 5B; arrowheads).

Single N cell spikes elicited only small EPSPs in cell 157 (Fig. 5C). N cells are not known for being involved in the local bend behavior18,28. But, nevertheless, this result might indicate a synaptic connection between N cells and the local bend IN. Thus, N cells might either have a more significant role for this behavior at higher touch intensities21,22 or the INs are involved in other functional networks in addition to the local bend network.

### Stimulus estimation results

**Figure 6:**
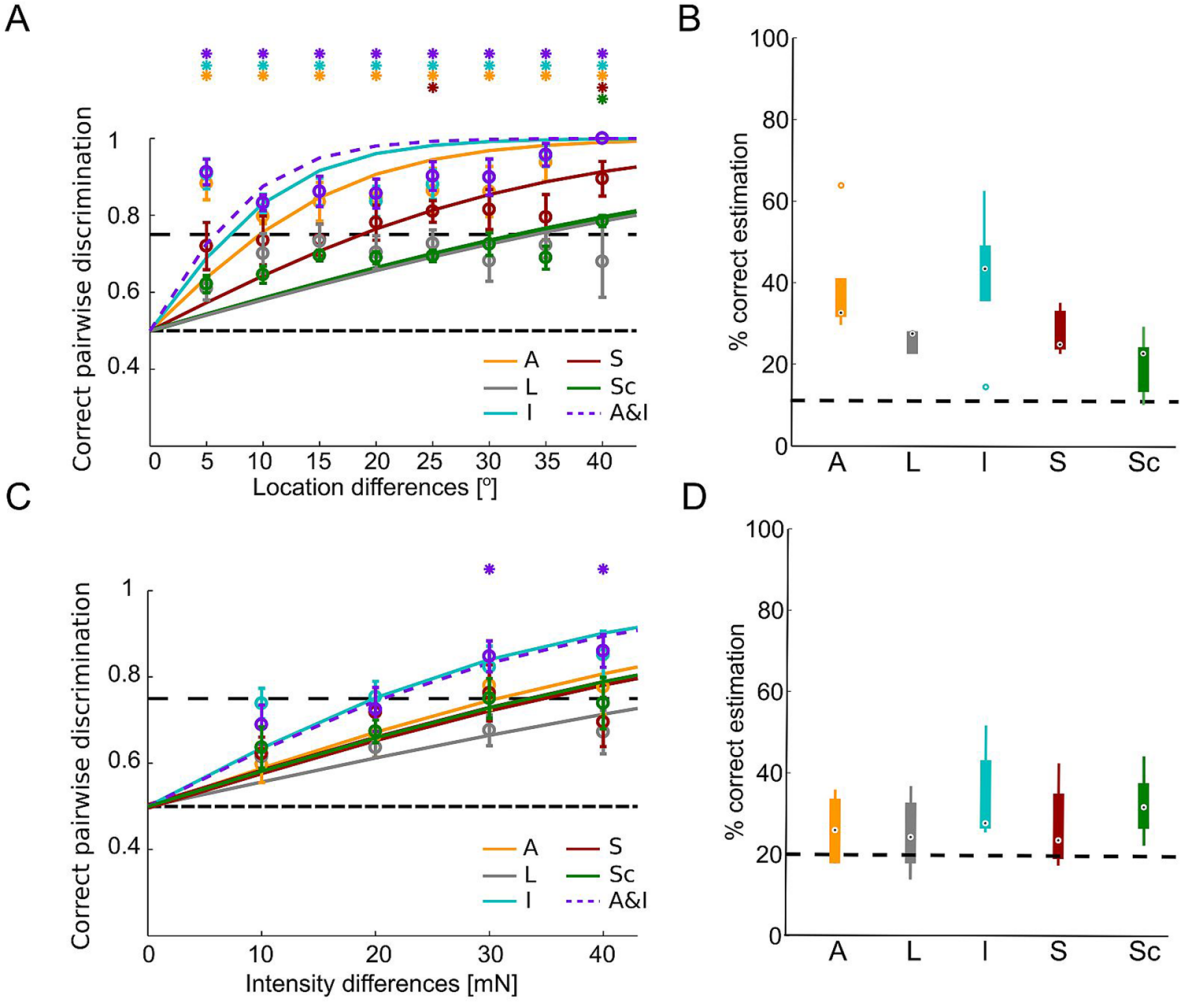
Stimulus estimation results for location and intensity of cell 157. (**A**) Pairwise discrimination (mean and SEM) for location differences between 5° and 40°, with stimulation centre 0°, for a 50 mN touch stimulus (N_cells_ = 5; N_animals_ = 4; see Fig. 3D–F). Black dashed lines show chance level (50%) and 75%-threshold. Asterisks indicate mean values, which are significantly (p < 0.05, t-test) above threshold. (**B**) Classification results of data corresponding to A. Black dashed line show chance level. Black dots mark median values in boxplots. A = Amplitude; L = Latency; I = Integral; S = Slope; Sc = Spikelet count. (**C**) Pairwise discrimination (mean and SEM) of intensities from 10 to 50 mN, resulting in minimal intensity difference of 10 mN and maximal difference of 40 mN, at 0° (N_cells_ = 7; N_animals_ = 7; see Fig. 4B–D). (**D)** Classification results corresponding to C.

To evaluate the information carried by the IN responses about the stimulus, we combined two complementary approaches: discrimination of stimulus differences and classification of all possible stimulus conditions (see *Methods*). Overall, responses of cell 157 allowed significant discriminations of very small (5°) touch location differences based on graded response features, with amplitude and integral performing similarly well (Fig. 6A). A combination of these two features did not improve the estimation significantly (Fig. 6A). As an additional response feature, we tested the spikelet count of cell 157. This feature did not yield a discrimination performance significantly higher than the 75% threshold (Fig. 6A). The classification of nine locations led to 43.75% (median) correct estimation for the integral of the cell 157 responses (Fig. 6B). The other response features also led to classification results well above chance level (Fig. 6B). Intuitively, the good estimation performance of the integral is not surprising, since this response feature depends on amplitude as well as slope and hence may reflect the IN-response shape most reliably.

We tested how strongly two touch intensities have to differ to be distinguishable based on the response features. A feature combination of integral with amplitude allows the detection of 30 mN intensity differences significantly above threshold (Fig. 6C). This result is in agreement with behaviourally determined detection thresholds^10^. The best classification result for five intensities (increment 10 mN) was obtained by the integral, yielding a median of 28% correct classification (Fig. 6D). The other response features led to percentages of correct classification even closer to the chance level of 20% (Fig. 6D). However, it should be kept in mind that the ability of leeches to discriminate stimulus intensities behaviourally is higher for low intensities and falls off linearly with rising intensities^10^.

## Discussion

Small neuronal systems can be used to investigate how information from sensory stimuli is translated into surprisingly accurate behavioural outputs. The local bend response is one of the fastest behaviours of the leech, with muscle movements starting only 200 ms after stimulus onset^10,18^. Furthermore, the precision of the animal’s ability to discriminate two touch locations is comparable to the human finger tip^1,10^. After investigating touch encoding by mechanosensory cells^21^, we studied here two IN types in the second layer of the neuronal network. Specifically, we aimed to reveal how these cells respond to tactile stimulation of the skin, if they are connected to all types of mechanosensory cells and whether it is possible to predict the presented stimulus from graded IN responses. The results indicate that in addition to P cells, T cells also modulated the IN responses. Preliminary data of N cell and INs suggest that all types of mechanosensory cells might project onto the same type of INs. Different response characteristics to tactile stimulation seen in different IN types suggest specialised filter and integration mechanisms within this behaviour.

### Interneuron responses to tactile stimulation

The receptive field of cell 157 (Fig. 3) fits in with the receptive fields of other local bend INs, as inferred by Lewis^19^. Most IN types are paired^5^ and receive inputs from more than one mechanosensory cell^5,11^. Consequently, INs may have broader receptive fields than the latter, suggesting a receptive field up to 360° whereas the mechanosensory cells innervate an area of about 180° of the circumference^11^. Thus, the receptive fields of an IN pair span the whole circumference of the segment with a huge overlap, while on the level of the mechanosensory cells, the same area is innervated by four (P cells) respectively six (T cells) cells^11^.

Responses of the INs depended also on touch intensity (Figs 4 and 6). Similar dependencies were found for mechanosensory cells^21,22^. It is not clear yet to what extent, INs of the leech are specialised in processing single touch properties or their combinations and whether multiplexing plays a similar role as found for mechanosensory cells^21^. In the stick insect, descending INs were found which were specialised for a single stimulus property, but additionally were activated by other stimulus properties^25^. The leech local bend network provides a good model system to further investigate the fundamental question of how combinations of relevant stimulus properties are processed at the IN level to elicit specific, accurate behavioural responses.

### Connections between mechanosensory cells and interneurons

Previous studies on the local bend behaviour focused on P cells and synaptic connections between this cell type and the local bend INs^4–8,18,26–28^, and the first assumption was that the local bend network could be represented as a simple feed-forward circuit^11^. However, more recent studies revealed more complex mechanisms within this circuitry, like lateral connections of mechanosensory cells and motor neurons^29–31^. T cells receive polysynaptic, mostly inhibitory input from P cells and N cells^29^, and P cells also form inhibitory polysynaptic chemical connections on other P cells in the same ganglion^30^. These lateral interactions on sensory cell level might play a role in localization of the local bend response^23,30^. Additionally, lateral inhibition among motor neurons and a widespread type of inhibition were also found, suggesting that the local bend network may use balanced excitation and inhibition for gain control^31^. To our knowledge, no excitatory connections from T to P cells or from N to P cells were found^29^. This is consistent with our own experience, in which we never saw effects in P cells when stimulating the other mechanosensory cell types electrically. For the polymodal N cells, it was found that high-frequency stimulation can cause potentiation of P cell synapses^23,32^. However, in our experiments N cells were never firing high frequencies^21,22^.

By labelling multiple cells in the leech nervous system, we found putative input sites of P and T cells to cell 157, as well as of T cells to cell 159 (Fig. 5A,B). Electrophysiological experiments confirmed these findings and showed that single N cell spikes also elicited EPSPs in cell 157 (Fig. 5C). Furthermore, we found that cell 157 was influenced by spikes of ipsi- as well as contralateral mechanosensory cells. This is in line with results shown by Lockery and Kristan^5^ for paired intracellular recordings of dorsal P cells and cell 157. Remarkably, the INs showed short response latencies that were slightly longer than the T cell and shorter than P cell response latencies^21^ (Figs 3 and 4). This strong T cell influence on the initiation of the IN response supports our previous findings^21,22^ clearly suggesting the involvement of T cells in the local bend network. Here, we did not explicitly test the kind of synaptic connection between cell 157 and the mechanosensory cells nor did we define the synaptic weight of single mechanosensory cells by evaluating the EPSPs based on the elicited number of mechanosensory spikes. Even though the cell-specific structural imaging might suggest monosynaptic coupling between the mechanosensory cells and cell 157 and cell 159, the connection could be monosynaptic or polysynaptic, electrical or chemical. This circuit needs to be characterized in more detail in future studies to discern correlations among stimulus properties, activity of mechanosensory cells and INs, and the behavioural muscle response.

The examined INs seem to use different strategies for combining mechanosensory cells input. Cell 157 tends to integrate EPSPs coming from all three types of mechanosensory cells with a long time constant (Fig. 2A). In contrast, responses of cell 159 seem to follow mainly the fast-adapting T cell responses, leading to shorter, more transient membrane potential fluctuations (Fig. 2B). These findings may indicate principal differences in the role of different INs in the network, e.g., integration versus coincidence detection of multiplexed information of several stimulus properties. Furthermore, previous findings may suggest an involvement of cell 157 and 159 in other behaviours: Briggman and colleagues^33,34^ used voltage sensitive dye recording (VSD) to investigate decision-making in the leech and found neurons that discriminated very early in time between the two behaviours of swimming and crawling^33,34^. The neurons were found in the region of the ganglion where cell 157 and 159 are located (Fig. 4C in Briggman *et al*.^33^; Fig. 5B in Briggman and Kristan^34^). Multifunctional INs relevant for several neuronal circuits were also described by Frady and colleagues^35^. The recent availability of double-sided VSD imaging could help to shed light on these multifunctional INs and to give an overview on the neuronal circuits in the whole ganglion^36^. Overall, the small system of the leech allows basic conclusions to be drawn about processing of information through a multi-layered network with a defined set of behavioural outputs.

### Estimation of stimulus properties based on graded signals

Most studies on neural coding and stimulus estimation have focused on the analysis of spike trains^37,38^. However, at the level of INs graded responses play a significant role in information processing, at least in invertebrate systems and also in vertebrate sensory systems like the retina^39^. Bipolar cells transfer the graded photoreceptor information to ganglion cells and this signal is modulated by retinal INs, horizontal and amacrine cells, solely through graded signals. De Ruyter van Steveninck and Laughlin^40^ concluded in a computational study that graded signals are specialised for accurate information processing over short distances.

In our study, features of graded responses were used to estimate underlying stimulus properties. Very small location and intensity differences could be discriminated based on responses of one IN type (cell 157) receiving input from three mechanosensory cell types (T, P, N) simultaneously. The IN responses decode the input of the mechanosensory cell population in a precise manner. The best and most reliable stimulus estimation results were obtained from the integral. The other response features, in particular the latency, yielded less reliable stimulus estimates and are more susceptible to stochastic membrane potential fluctuations and spikelets. Emergence and origin of spikelets were investigated in different species and sensory systems^24,41–44^ but the role of spikelets in neuronal information processing still needs to be investigated. However, Lockery and Kristan^5^ did not find a correlation between these small action potentials and motor neuron spikes in the leech. In agreement with these findings, we found in this study that spikelet counts and interspikelet intervals (not shown) did not improve the stimulus estimation and yielded results in the range of IN response latency and the slope (Fig. 6).

The local bend network appears to be a small but complex neuronal circuit^29–31^. This study suggests that in addition to P cells, T cells and possibly N cells provide input to the network. The different response patterns of the IN types may indicate specialisation involved in multiplexed population coding as suggested by Pirschel and Kretzberg^21^. Local bend INs might process the relative latencies as coincidence detectors and consequently decode the touch location. Or they might merge, as slow integrators, the spike counts for decoding the touch intensity. Moreover, the simple nervous system of the leech processes information in the form of spike trains of mechanosensory cells which result in graded signals of INs, which are translated back into spike trains by motor neurons. Thus, this animal model allows insights into general principles of sensory coding up to behavioural importance of multifunctional INs and distinct information processing mechanisms.

## Materials and Methods

### Physiology

The leeches (adult *Hirudo verbana*; hermaphrodites; distributed by: bbez, Biebertal, Germany) weighed 1–2 g and were kept at room temperature in ocean sea salt at 1:1000 dilution with purified water. Body-wall preparation (Fig. 1), mechanical stimulation and electrophysiological recordings were carried out as previously described in detail by Pirschel and Kretzberg^21^. In total, 39 body-wall preparations were included in this study. Throughout, the directional terms ‘left’ and ‘right’ are from the experimenter’s perspective^1,21^ (Fig. 1). Touch locations to the left of the ventral midline (defined as 0°) were denoted as negative and to the right as positive numbers of degrees (Fig. 1). We performed intracellular recordings from mechanosensory cells and INs of the local bend network, while stimulating the skin mechanically^21,22^. The local bend INs were identified according to morphological and physiological properties described by Lockery and Kristan^5^. We recorded from mechanosensory cells with ventral receptive fields: ventral P and T cells and lateral (polymodal) N cells^14–17^ (Fig. 1). The mechanosensory cell types were identified based on their properties described in previous investigative studies^3,10,14–18^ as well as their responses to tactile stimulation.

Mechanical stimulation was provided using the Dual-Mode Lever Arm System^1,10,21^ (Aurora Scientific, Ontario, Canada, Model 300B; poker tip size 1 mm^2^) at the 3^rd^ annulus of segment 10 (identified by location of the sensilla^17^). We present results for touch locations from −20° to +20° in 5° steps for a touch intensity of 50 mN (N_cells_ = 5, consisting of 3 left, 2 right cells; N_animals_ = 4; Figs 2 and 3). The stimulus intensity varied between 10 and 100 mN and was presented for low (<50 mN) and high (50–100 mN) intensity groups with a touch duration of 200 ms^1,18^ (Fig. 4). All combinations of stimulus properties were presented 10 times in a pseudo-randomized order. Location estimation was done across five experiments with cells 157 which were stimulated at locations −20° to +20° in 5° steps with 50 mN stimulus intensity. For intensity estimation, cells 157 (N_cells_ = 7, consisting of 4 left, 3 right cells; N_animals_ = 7) were stimulated with intensities between 10 and 50 mN at location 0°.

To identify synaptic connections between INs and mechanosensory cells, intracellular recordings of the INs were obtained, while mechanosensory cells were stimulated intracellularly by current pulses. The pulse strength was chosen between 1 and 2.5 nA, based on the spike thresholds of the mechanosensory cells, and lasted for 50 ms. For cell 157, we analysed 29 paired recordings with respect to their location in the ganglion (definition see Lockery and Kristan^5^): 5 ipsi- and 5 contralateral P cells; 3 ipsi- and 6 contralateral N cells; and 4 ipsi- and 6 contralateral T cells. For cell 159, one ipsilateral combination with each mechanosensory cell type was considered.

The datasets generated and analysed during the current study are available from the corresponding author on reasonable request.

### Morphology

For anatomical studies, isolated ganglia of the 10^th^ segment were used. To visualise cell morphologies and points of contact, we used the same approach as described previously in Kretzberg *et al*.^22^. Briefly, sharp glass electrodes (20–40 MΩ) were used to fill INs and mechanosensory cells with 10 mM Alexa-dyes (Invitrogen, Karlsruhe, Germany) and/or 2% Neurobiotin (Vector Labs, Peterborough, UK), both diluted in 200 mM KCL. Dyes were iontophoretically injected to the cell either with positive (Neurobiotin) or negative (Alexa) currents (2–4 nA, 500 ms, 1 Hz, 30–60 min). Immediately after dye injection cells were fixed in 4% PFA (Sigma, Munich, Germany) for up to 1 hour. The preparation was then rinsed in 0.1 M PBS (6 × 10 min) and was incubated in 1:1000 Streptavidin (Vector Labs)/PBS/0.5% Triton-X overnight at 4 °C. After a 6–10 minute rinse in PBS, the ganglion was embedded with VectaShield (Vector Labs) on a microscope slide for confocal microscopy. For multiple dye fills (see Fig. 5), Alexa-dyes as well as Neurobiotin were used.

### Analysis

Spikes of mechanosensory neurons elicit postsynaptic potentials (PSPs) in the IN soma. Response traces were filtered offline with a Notch filter (Notch frequency: 50 Hz; Matlab Filter Design Toolbox, MathWorks, Natick, MA, USA). Resting potentials of the IN were computed as the averaged potential of one second before stimulation onset. To define reference values for the interneuron responses (e.g., start time of the PSP, time of the maximum amplitude), the neuronal response was averaged in a sliding time window of 5 ms (steps of 1 ms) in order to smooth out high-frequency fluctuations from recording noise. The start time of the IN response was identified as the first-time window when the membrane potential exceeded the resting potential by two-folds of standard deviation. Spikelets were defined by means of amplitude (threshold = 2 mV depolarization) and duration (time window = 15 ms): a spikelet was detected when the membrane potential depolarized by the threshold value and fell by half of the peak amplitude within the time window.

The following features quantified the IN responses (Fig. 1D):

L: Latency [ms], time between stimulus onset and starting point of the PSP.

S: Slope [mV/ms], inclination of the signal from start of PSP (≙ latency) to 30 ms post start time.

I: Integral [mV × ms], area, from start of PSP to 200 ms post start time, between the graded signal and the resting potential.

A: Maximal amplitude [mV], difference between the PSP maximum and the resting potential.

Sc: Spikelet count, during start of stimulation to 200 ms post start time.

### Stimulus estimation

Our method provides an insight into possible encoding strategies, which may be used by the neuronal system. Following our preceding study^21^, we used two different estimation approaches, a pairwise discrimination and a classification, both based on the maximum-likelihood method^45^ with a leave-one-out validation^46^.

Basically, the maximum likelihood method predicts the presented stimulus that most likely elicited the neuronal response. For each neuronal response, the response features amplitude, slope, response latency, and spikelet count were determined. The presented stimulus was characterized by the value of the varied stimulus property, i.e. touch location or intensity. The estimation was expected to reveal specific response features that encoded the presented stimulus property best. To enable a fair and reliable comparison of the different response features, we used response feature classes containing ranks rather than the raw data for the stimulus estimation^21^. For feature combinations, the feature ranks were combined to yield one data set^21^. This rank-based approach simplified the comparison of response features having different statistical properties and different combination of features^21^.

The leave-one-out validation was used for the definitions of test data and training data: each recording trial was used once as test data, while all other trials comprised the training data set. For the training data set, it was known which stimulus condition elicited the response. Therefore, the training data set was used to determine probability distributions of response feature classes for each stimulus condition. This knowledge provided the basis to determine the stimulus condition that had the highest probability (maximum likelihood) of eliciting the response feature value observed in the test data^21^. If this result, the estimated stimulus condition, matched the actual stimulation that elicited the response in the test data, the trial was counted as correct estimation. This procedure was repeated for each recording trial, leading to a percentage of correct stimulus estimations. Based on this approach, the pairwise discrimination^1,21^ allows two stimuli to be discriminated based on their neuronal responses, resulting in minimum distinguishable differences of intensities or locations. Results are represented as mean values with standard error of the means (SEM) and fitted with a logistic function. Chance level of pairwise discrimination is 0.5 and discrimination threshold is defined as 0.75, which corresponds to 75% correct estimation^1,47^. The classification^21^ compares the complete set of stimulus conditions and indicates how well these stimuli could be distinguished. For the estimation of the touch location, we used locations between −20° and +20°, which results in nine possible stimulus conditions and, since in our data set all stimuli were presented with equal probability, a chance level of 11.11%. The chance level for this method was defined as 100/N %, where N represents the number of stimulus conditions. Results are given in %-correct and displayed in boxplots (Fig. 6). Black dots mark the median values and box edges the 25th (*q*_1_) and 75th (*q*_3_) percentiles. Whiskers show minimum and maximum data values. Outliers are defined by (per standard definition of Matlab boxplot function):

$${\rm{x}} > {q}_{3}+1.5({q}_{3}-\,{q}_{1})\,{\rm{or}}\,{\rm{x}} < {q}_{1}-\,1.5({q}_{3}-{q}_{1}),$$

and plotted as individual dots.

### Significance tests

Significant dependencies of response features on stimulus properties were identified with the Friedman test^48^, a non-parametric version of the one-way analysis of variance (ANOVA). The Kolmogorov-Smirnov test was used to investigate significant membrane potential changes of INs in response to spikes of mechanosensory cells.

To define whether the pairwise discrimination results are significantly above the performance threshold of 75%, we applied a one-tailed t-test compared to 0.75. The classification results were tested with the Wilcoxon rank sum test (equivalent to a Mann-Whitney U-test)^48,49^, with null hypothesis that the two independent data sets are from identical distributions with equal medians. Tests were computed using the Matlab Statistics Toolbox (MathWorks, Natick, MA, USA). For more detailed description see Pirschel and Kretzberg^21^.
